# Effects of Psychopathy on Neurocognitive Domains of Impulsivity in Abstinent Opiate and Stimulant Users

**DOI:** 10.3389/fpsyt.2021.660810

**Published:** 2021-06-09

**Authors:** Elena Psederska, Nicholas D. Thomson, Kiril Bozgunov, Dimitar Nedelchev, Georgi Vasilev, Jasmin Vassileva

**Affiliations:** ^1^Bulgarian Addictions Institute, Sofia, Bulgaria; ^2^Department of Cognitive Science and Psychology, New Bulgarian University, Sofia, Bulgaria; ^3^Division of Acute Care Surgical Services, Department of Surgery, Virginia Commonwealth University Health, Richmond, VA, United States; ^4^Department of Psychology, University of Durham, Durham, United Kingdom; ^5^Institute for Drug and Alcohol Studies, Virginia Commonwealth University, Richmond, VA, United States; ^6^Department of Psychiatry, Virginia Commonwealth University, Richmond, VA, United States

**Keywords:** opioid use disorder, stimulant use disorder, psychopathy, impulsivity, decision-making, response inhibition

## Abstract

**Background:** Psychopathy and substance use disorders (SUDs) are both characterized by neurocognitive impairments reflecting higher levels of impulsivity such as reward-driven decision-making and deficient inhibitory control. Previous studies suggest that psychopathy may exacerbate decision-making deficits, but it may be unrelated to other neurocognitive impairments among substance dependent individuals (SDIs). The aim of the present study was to examine the role of psychopathy and its interpersonal-affective and impulsive-antisocial dimensions in moderating the relationships between dependence on different classes of drugs and neurocognitive domains of impulsivity.

**Method:** We tested 693 participants (112 heroin mono-dependent individuals, 71 heroin polysubstance dependent individuals, 115 amphetamine mono-dependent individuals, 76 amphetamine polysubstance dependent individuals, and 319 non-substance dependent control individuals). Participants were administered the Psychopathy Checklist: Screening Version (PCL:SV) and seven neurocognitive tasks measuring impulsive choice/decision-making (Iowa Gambling Task; Cambridge Gambling Task; Kirby Delay Discounting Task; Balloon Analog Risk Task), and impulsive action/response inhibition (Go/No-Go Task, Immediate Memory Task, and Stop Signal Task).

**Results:** A series of hierarchical multiple regressions revealed that the interpersonal-affective dimension of psychopathy moderated the association between decision-making, response inhibition and both amphetamine and heroin dependence, albeit differently. For amphetamine users, low levels of interpersonal-affective traits predicted poor decision-making on the Iowa Gambling Task and better response inhibition on the Stop Signal task. In contrast, in heroin users high interpersonal-affective psychopathy traits predicted lower risk taking on the Cambridge Gambling Task and better response inhibition on the Go/No-Go task. The impulsive-antisocial dimension of psychopathy predicted poor response inhibition in both amphetamine and heroin users.

**Conclusions:** Our findings reveal that psychopathy and its dimensions had both common and unique effects on neurocognitive function in heroin and amphetamine dependent individuals. Our results suggest that the specific interactions between psychopathy dimensions and dependence on different classes of drugs may lead to either deficient or superior decision-making and response inhibition performance in SDIs, suggesting that psychopathy may paradoxically play a protective role for some neurocognitive functions in specific subtypes of substance users.

## Introduction

### Impulsivity and Substance Use Disorders

Impulsivity, defined as a “predisposition toward rapid, unplanned reactions to internal or external stimuli without regards to the negative consequences of these reactions” (1) is considered a key etiological factor in current conceptualizations of substance use disorders (SUDs) (2). Deficits in impulse control are considered both as vulnerability factors that increase the risk of initiation and maintenance of SUDs (3, 4), as well as consequences of chronic drug use reflecting long-term neuroadaptive changes in the brain linked to specific neurocognitive impairments (5, 6). Despite the strong associations of impulsivity with SUDs, recent advances in the literature have drawn attention to the multifactorial nature of impulsivity and the heterogeneity of SUDs, suggesting that specific impulsivity dimensions might be differentially implicated in distinct types of SUDs and in different stages of the addiction cycle (2, 4, 7).

Impulsivity is a multidimensional construct comprised of a variety of characteristics reflecting the personality dimensions of trait impulsivity, as well as a number of neurobehavioral manifestations, reflecting more fluctuating neurocognitive dimensions of state impulsivity (8). Trait impulsivity is a stable personality dimension, widely acknowledged as a general risk factor for SUDs (9), which is usually measured by self-report questionnaires such as the Barratt Impulsiveness Scale-11 [BIS-11; (10)] and the UPPS Impulsive Behavior Scale [UPPS; (11)]. Trait impulsivity is considered to be on a continuum between lower, more adaptive levels and higher, more extreme and maladaptive levels, which feature prominently in externalizing psychiatric disorders that originate in childhood and are commonly comorbid with SUDs, such as attention deficit hyperactivity disorder (ADHD), oppositional defiant disorder (ODD), conduct disorder, and antisocial personality disorder (ASPD) (12). In contrast to trait impulsivity, neurocognitive dimensions of impulsivity are more fluctuating and dependent on environmental influences and the current state of the individual (9). Therefore, neurocognitive domains of impulsivity reflect more imminent risk and are typically measured in the laboratory with performance-based computerized tasks.

Neurocognitive impulsivity is additionally subdivided into two broad domains: impulsive action, involving deficits in rapid response inhibition (13) and impulsive choice, indicating deficits in decision-making (14). This distinction is supported by findings from preclinical studies, which show that impulsive choice and impulsive action are differentially involved in distinct stages of the addiction cycle and are mediated by different neural circuits (15). Impulsive action reflects response disinhibition and is typically measured by Stop Signal Tasks [SST; (16)], which examine the ability to cancel an already initiated motor response, and/or Go/No-Go type of paradigms (17, 18), measuring the ability to inhibit a prepotent or dominant behavioral response. Impulsive choice reflects a reward-driven decision-making style associated with higher risk-taking and preference for immediate over delayed rewards. Common tasks of impulsive choice include delay discounting tasks (19, 20) such as the Monetary Choice Questionnaire [MCQ; (21)] and simulated gambling tasks measuring sensitivity to risk and reward, such as the Iowa Gambling Task [IGT; (22)] measuring decision-making under ambiguity or the Cambridge Gambling Task [CGT; (23)] and the Balloon Analog Risk Task [BART; (24)], measuring decision-making under risk.

### Neurocognitive Impulsivity in Substance Use Disorders

Impairments in neurocognitive impulsivity have long been implicated in SUDs. Increased response disinhibition and aberrant decision-making are some of the most common findings in people with SUDs (23, 25–31). Deficits in neurocognitive dimensions of impulsivity have gained increased research interest in the addiction literature as predictors of drug initiation and poor treatment outcomes. Studies reveal that higher delay discounting and compromised decision-making are predictive of post-treatment relapse and can negatively affect one's ability to achieve and maintain abstinence from substance use (32–37). Although response disinhibition on Stop Signal and Go/No-Go tasks has not been consistently related to treatment retention and abstinence (34, 35), it has proven to be among the most reliable predictors of drug use initiation (38–41).

Though individuals with SUDs manifest marked impairments on virtually all tasks of impulsive choice and impulsive action (23, 25, 28, 30, 31), recent studies suggest that the type of deficits demonstrated by individuals with SUDs might also be affected by the unique properties of the type of substance they are using. In line with the precision medicine approach, current models of addiction emphasize the increasing need for identifying substance-specific personality and neurocognitive risk profiles that reflect the specific psychopharmacological effects of different classes of drugs and the distinct positive and negative reinforcement mechanisms implicated in different types of SUDs (2, 42, 43). Research increasingly reveals differences in neurocognitive dimensions of impulsivity in individuals with different SUDs, such as stimulant and opioid use disorder. Although there is accumulating evidence for impaired response inhibition on impulsive action tasks in individuals with both stimulant- (28, 31, 44–46) and opioid use disorders (47–49), studies directly comparing opiate and stimulant users reveal that stimulant users are characterized by more pronounced response inhibition deficits than opiate users (31, 50). Studies investigating impulsive choice in individuals with stimulant and opioid use disorders have yielded somewhat mixed findings. Some studies have shown that individuals who preferentially use stimulants are characterized by more impulsive decision-making than opiate users (20, 23, 50, 51), whereas others have failed to find any performance differences between stimulant and opiate users (31, 52, 53). Machine-learning approaches also reveal that heroin and amphetamine dependence are characterized by unique substance-specific neurocognitive impairments (54, 55), with heroin dependence uniquely predicted by impaired decision-making, lower risk-taking and intact response inhibition, whereas amphetamine dependence was predicted by higher delay discounting and longer reaction times (54).

However, there are several methodological limitations that limit the conclusions that can be drawn from previous studies in the field. Polysubstance use is one of the most significant confounds in studies aiming to dissociate the specific effects of different classes of drugs. With few exceptions (7, 52, 54), most studies examining differences in neurocognitive impulsivity between opiate and stimulant users are based on samples of polysubstance users whose drug of choice was either opiates or stimulants (23, 31, 50, 51, 53). Another methodological limitation is related to differences in the length of abstinence across studies of neurocognitive function in substance users. The majority of neurocognitive studies on impulsivity explore the effects of chronic substance use or the effects of early remission (<12 months) (20, 23, 25, 28, 31, 45–47, 50, 51, 53). A few studies have focused on elucidating the effects of protracted abstinence (>12 months) on different dimensions of neurocognitive impulsivity (7, 52, 53, 56–58). Differences in the length of abstinence (early vs. protracted) of participants with SUDs may explain some of the conflicting findings in the literature, as some neurocognitive deficits have been shown to recover with abstinence (59–61). However, few neurocognitive studies in SDIs have addressed the protracted abstinence stage of the addiction cycle. Finally, neurocognitive studies often fail to control for the confounding effects of externalizing traits among people with SUDs, such as antisocial and psychopathic traits, which are characterized by similar neurocognitive impairments as those observed in substance users and may further exacerbate neurocognitive impairments in SDIs.

### Effects of Psychopathy on Neurocognitive Impulsivity in Substance Users

Psychopathy is a personality disorder characterized by a cluster of personality and behavioral traits, which fall into two factors. Factor 1 is characterized by affective (e.g., callousness, lack of remorse) and interpersonal traits (e.g., manipulativeness, superficial charm), whereas Factor 2 consists of lifestyle (e.g., impulsivity, irresponsibility) and antisocial traits (e.g., early behavior problems, poor behavioral controls) (62). This distinction is reflected in the Psychopathy Checklist-Revised [PCL-R; (63, 64)], the most widely used instrument for measuring psychopathy, which differentiates between interpersonal-affective and impulsive-antisocial features of psychopathy (65, 66), closely resembling the traditional distinction between primary and secondary psychopathy (67–69). Studies with the PCL-R reveal that Factor 1 is uniquely related to lower levels of anxiety and impulsivity, whereas Factor 2 is associated with negative emotionality, impulsivity, and substance misuse (63, 70, 71).

Psychopathy often co-occurs with SUDs (72–74) and is associated with a variety of negative outcomes in people with SUDs, including high treatment attrition, substance use during treatment, high relapse rates, and increased risk for post-treatment violent offending (73, 75–77). Studies using machine-learning approaches reveal that psychopathy is the highest and the only common predictor of dependence on different classes of drugs, including heroin, amphetamine, cannabis, nicotine, and alcohol (54, 55). This suggests that psychopathy may be an important diagnostic marker for SUDs, regardless of drug class.

Psychopathy has been associated with impairments in neurocognitive domains of impulsivity, similar to those observed in individuals with SUDs. With few exceptions (78, 79), most studies on impulsive choice in psychopathy have found that psychopathic individuals manifest suboptimal decision-making, associated with risky decision-making style and inability to learn from feedback (80–86). Results are less consistent in the impulsive action domain, with some studies reporting higher response disinhibition (87–90), whereas others suggest intact or even superior response inhibition in psychopathic individuals (87, 91–93). Inconsistencies across findings may be explained by the heterogeneity of psychopathy, which has not been addressed by the majority of studies, which are typically based on PCL total sum scores that do not take into account the distinction between interpersonal-affective and impulsive-antisocial aspects of psychopathy. Focusing exclusively on total sum scores may lead to conflicting results and conceal important differential relationships that could deepen our understanding of psychopathy (94). Studies that have addressed the distinction between the interpersonal-affective and impulsive-antisocial dimensions of psychopathy reveal that only Factor 2 (impulsive-antisocial) is related to impulsive choice, manifested by risky and less advantageous decision-making (80, 95, 96). With regards to impulsive action, studies demonstrate that higher scores on PCL-R Factor 2 and lower scores on Factor 1 were related to poor response inhibition, suggesting that the affective-interpersonal aspects of psychopathy may in fact exert some protective effects on neurocognitive functioning (97, 98).

Given that both psychopathy and SUDs are associated with neurocognitive deficits in impulsivity, it has been suggested that their co-occurrence may increase some impulse-control deficits in individuals with SUDs (86). In two related studies, Vassileva et al. (86, 92) examined differences in various neurocognitive domains of impulsivity in psychopathic and non-psychopathic mono-substance dependent (“pure”) heroin users. Findings revealed that comorbid psychopathy exacerbated decision-making deficits in heroin dependent individuals (86), but psychopathy was unrelated to delay discounting and response inhibition in this population (92). However, the role of psychopathy and its dimensions on neurocognitive functioning in SUDs is still not well-understood and has been particularly understudied among individuals dependent on different classes of drugs and in different stages of the addiction cycle. This is an important line of inquiry as Factor 1 and 2 may be differentially related to neurocognitive functioning and impulsivity (99, 100), which could in turn influence the associations between SUDs and neurocognitive function.

The aim of the current study was to examine if psychopathy and its dimensions moderate the relationships between addiction to different classes of drugs (stimulants vs. opiates) and neurocognitive domains of impulsivity (impulsive choice and impulsive action) in substance users in protracted abstinence.

## Materials and Methods

### Participants

Participants were recruited from a larger study on impulsivity among substance users in Bulgaria via flyers placed at substance abuse clinics, therapeutic communities, social venues, as well as through the study's web page and Facebook page. Participants were initially screened via telephone on their medical and substance use histories. All participants had to meet the following inclusion criteria: (1) age between 18 and 50 years, (2) Raven's Progressive Matrices (101) estimated IQ higher than 75; (3) minimum of 8th grade education; (4) being able to read and write in Bulgarian; (5) HIV-seronegative status; (6) negative breathalyzer test for alcohol and negative urine toxicology screen for amphetamines, methamphetamines, cocaine, opiates, methadone, cannabis, benzodiazepines, barbiturates, and MDMA. Exclusion criteria included history of neurological illness, head injury with loss of consciousness of more than 30 min, and history of psychotic disorders and/or use of antipsychotic medication.

Participants included 693 individuals (64% male), with a mean age of 28.57 years (*SD* = 7.09). Three hundred seventy-four participants (74.1% male) had a DSM-IV history of substance dependence, of whom 183 were dependent on heroin (77% male) (112 mono-dependent, 71 polysubstance dependent) and 191 were dependent on amphetamines (71.2% male) (115 mono-dependent, 76 polysubstance dependent). The majority of participants with a history of substance dependence (69%) were in protracted abstinence at the time of testing (i.e., full sustained remission for more than 12 months by DSM-IV criteria) (102). In addition, 319 participants (53% male) had no past or current history of abuse or dependence on any substance, of whom 62 were non-substance dependent siblings of heroin users (44% male), and 48 were non-substance dependent siblings of amphetamine users (40% male).

### Procedures

The study was approved by the Institutional Review Boards of Virginia Commonwealth University and the Medical University in Sofia on behalf of the Bulgarian Addictions Institute. Subjects who met inclusion criteria were invited to participate in the study. All participants gave written informed consent. Abstinence from alcohol and drug use at the time of testing was verified by breathalyzer test (Alcoscan AL7000) and urine toxicology screen for amphetamines, barbiturates, benzodiazepines, cannabis, cocaine, MDMA, methadone, methamphetamines, and opiates. All participants were HIV-seronegative, determined by rapid HIV testing.

Testing was conducted by an experienced team of trained psychologists at the Bulgarian Addictions Institute in Sofia, Bulgaria. Data were collected in two sessions of approximately 4 hours each, conducted on two separate days. The assessment battery included a combination of clinical interviews, self-report questionnaires and computer-based neurobehavioral tests. The first session included assessment of substance use disorders, externalizing psychopathology (e.g., psychopathy, antisocial personality disorder, ADHD) and intelligence. The second session included completion of neurocognitive tasks and self-report measures of externalizing and internalizing personality traits and disorders (e.g., impulsivity, sensation seeking, depression, alexithymia). Participants were paid a total of 80 Bulgarian leva (approximately 50 USD) for participation in the study.

### Measures

#### Assessment of SUDs and Psychopathy

Substance dependence was assessed with the *Structured Clinical Interview for DSM-IV—Substance Abuse Module* [SCID-SAM; (103)]. The SCID-SAM is a semi-structured clinical interview designed to determine whether an individual meets criteria for any SUD (alcohol-, cannabis-, stimulant-, hallucinogen-, opioid use disorders) according to the DSM-IV (102). Raters assess the presence of DSM-IV symptoms of substance abuse and dependence using a three-point scale (0 = not present, 1 = subthreshold, 2 = present). A diagnosis of substance dependence is made if the participant displayed three (or more) of the seven substance dependence criteria within a 12-month period. A symptom count of the number of criteria met for heroin- and amphetamine dependence (range 0–7) was used as the main SUD index in the analyses.

*The Psychopathy Checklist: Screening Version* [PCL:SV; (104)], an abbreviated version of the Psychopathy Checklist–Revised [PCL-R; (63)] was used to measure psychopathy. The PCL:SV consists of a semi-structured interview, which involves the assessment of 12 characteristics of psychopathy scored on a 3-point rating scale (0 = absent, 1 = somewhat present, 2 = definitely present). The PCL:SV is comprised of two factors. Factor 1 consists of six items reflecting the interpersonal and affective characteristics of psychopathy (grandiosity, manipulativeness, lack of empathy, lack of remorse), while the remaining six items from Factor 2 measure impulsive and antisocial behaviors (impulsivity, irresponsibility, poor behavioral controls, antisocial behavior in adolescence and adulthood). Items reflecting interpersonal-affective (Factor 1) and impulsive-antisocial (Factor 2) characteristics of psychopathy were summed to provide a total factor scores ranging from 0 to 12 points for each psychopathy dimension.

The semi-structured interview for the PCL:SV was conducted by researchers who were initially trained by the senior author, who is the author of the Bulgarian version of the PCL-R with its publisher Multi Health Systems. Additional training and supervision were further provided by two of the co-authors, who had participated in formal training workshops led by Robert Hare, the author of the PCL instruments. In line with earlier findings (105), the PCL:SV showed good internal consistency for its total score (α = 0.89) and its two factors (α = 0.78, and α = 0.85) in the current sample.

#### Neurocognitive Measures of Impulsivity

##### Measures of Impulsive Choice

*Iowa Gambling Task* [IGT; (22, 106)] measures decision-making under uncertainty and requires learning by trial-and-error. Examinees are presented with four decks of cards and instructed to select cards to maximize earnings. Decks A and B are associated with higher rewards but also higher occasional penalties. Selecting from Decks C and D yields lower rewards and lower occasional penalties and is a more advantageous long-term strategy. The performance measure used was the “net score” (IGT Net score), reflecting the total number of advantageous choices minus the total number of disadvantageous choices.

*Cambridge Gambling Task* [CGT; (23)] assesses risky decision-making, which does not involve learning. Examinees are presented with 10 boxes colored red or blue and are asked to guess whether a token is hidden under a red or a blue box. The ratios of red:blue boxes vary from 1:9 to 9:1 in pseudorandom order. Participants earn points based on correct performance. The second phase of the task asks participants to gamble points based on the confidence of their decisions, by selecting from an array of bets ranging from 5 to 95% of their earned points, presented in ascending and descending order. Two performance indices were used in the analyses: (1) Quality of decision-making (CGT Quality of decision-making), reflecting the tendency to bet on the more likely outcome; and (2) Risk taking (CGT Risk taking), the average number of points scored after the most probable result has been selected.

*Monetary Choice Questionnaire* [MCQ; (21)] was used to measure delay discounting. The questionnaire consists of 27 choices between smaller rewards available on the day of testing and larger rewards available from 1 week to 6 months in the future, thereby capturing the tendency to discount rewards that are delayed in time. The 27 questions were grouped in one of three categories based on the approximate magnitudes of the delayed rewards: small ($25–35), medium ($50–60) and large ($75–85). Analyses utilized the discount-rate parameter k, calculated using the hyperbolic discount function V = A/[1 + kD], where V is the value of reward A available at delay D. Two performance indices were used in the analyses: (1) the overall temporal discounting rate (i.e., MCQ Overall k); (2) the temporal discounting rate of small magnitude rewards (i.e., MCQ Small k), which typically has the highest effect sizes from the three reward magnitudes. We used the log transformed values of both discounting rates due to the non-normal distribution of MCQ scores in our sample.

*Balloon Analog Risk Task* [BART; (24)] is a decision-making task assessing risk-taking behavior. The participant is presented with a balloon on the computer screen, along with a balloon pump, a button for collecting the monetary rewards earned by pumping the balloon, a temporary bank, and a permanent bank, where the collected money from each balloon are kept. The task consists of a total of 30 balloons (trials) presented sequentially one at a time. At any point during each trial, the examinee can stop pumping the balloon and click the button to collect the money, which transfers the earnings accumulated from that balloon to the permanent bank. In contrast, when a balloon explodes, the balloon disappears, the money in the temporary bank is lost for that trial, and the next trial begins. The adjusted average number of pumps on unexploded balloons (BART Pumps adjusted average) was used as a measure of risk-taking, with higher scores indicative of greater risk-taking propensity.

##### Measures of Impulsive Action

*Go/No-Go Task* [GNGT; (18)] is a measure of response inhibition where a series of two-element visual stimuli arrays are presented on a screen for 500 ms and examinees are instructed to respond when the two elements are identical (“Go”) and to inhibit responding when the stimuli are discrepant (“No-Go”). On “No-Go” trials, the position of the inhibitory element is random, requiring the examinee to scan both elements. Errors of commission/false alarms (GNG False alarms) were used as an index of impulsivity in the regression analyses.

*Immediate Memory Task* [IMT; (17)] is a modified continuous performance task with higher complexity and sensitivity. A series of five-digit numbers are shown on a computer screen for 500 ms each, with examinees instructed to respond only if a stimulus is identical to the preceding one. Errors of commission (i.e., false alarms), measuring incorrect responding to a non-target stimulus (IMT Commission errors) were used as an index of impulsivity.

*Go Stop Task* [SST; (107)] is a stop-signal paradigm, which presents examinees with a series of five-digit numbers displayed for 500 ms each. Examinees are instructed to respond when a stimulus is identical to the previous display (“Go”) and to withhold responding when the stimulus matches, but then changes color from black to red (“Stop”). Stop signals occurred at 50, 150, 250, and 350 ms intervals after the appearance of the target “go” stimulus. The performance measure used in the analyses was the 150 ms inhibition ratio (SST 150 ms inhibition), calculated by dividing the failures to inhibit a response on “Stop trials” by correct detections on “Go trials” at the 150 ms stop-signal delay, which is the index most commonly used in the literature (107). Higher scores reflect better inhibition or lower impulsivity.

### Data Analytic Plan

Our main goal was to examine the moderating role of the two psychopathy dimensions on neurocognitive domains of impulsivity in heroin and amphetamine users. First, descriptive statistics and group differences in demographic characteristics, psychopathy scores and indices of impulsive choice and impulsive action were performed. Second, a series of hierarchical multiple regressions were conducted to examine the moderating role of psychopathy dimensions on the relation between substance dependence (heroin and amphetamine) and neurocognitive function (impulsive choice and impulsive action). All regressions followed the same steps. Step 1 included biological sex (1 = male, 2 = female), Raven's estimated IQ, heroin dependence symptoms, and amphetamine dependence symptoms. Step 2 added Factor 1 (interpersonal-affective) and Factor 2 (impulsive-antisocial) of psychopathy. Step 3 included the interaction terms between heroin dependence and psychopathy factors, and amphetamine dependence and psychopathy factors. All tests were conducted using an alpha of 0.05. Significant interactions were probed using simple slopes analysis (108).

## Results

### Descriptive Statistics and Group Differences

Group differences in demographic characteristics were examined using ANOVA. There were significant differences in age [*F*_(2, 689)_ = 41.92, *p* < 0.01], estimated IQ [*F*_(2, 690)_ = 5.90, *p* < 0.01] and years of education [*F*_(2, 687)_ = 29.46, *p* < 0.01] across groups. Tukey's *post-hoc* comparisons showed that amphetamine users were significantly younger than the two other groups, followed by control participants and heroin users (*p*s <0.01) With regards to estimated IQ, both control participants and amphetamine users scored higher than heroin users (*p*s < 0.05). In addition, control participants reported higher education as compared to both substance dependent groups (*p*s < 0.01). Group differences in substance use variables were examined using Independent Sample *t*-test. Amphetamine dependent individuals had lower length of abstinence [*t*_(280)_ = 5.10, *p* < 0.01] and lower symptoms count [*t*_(372)_ = 9.82, *p* < 0.01] compared to heroin dependent individuals. Group differences in indices of psychopathy and neurocognitive domains of impulsivity were examined using ANOVA followed by Tukey's *post-hoc* comparisons. There were significant group differences in both interpersonal-affective [*F*_(2, 690)_ = 173.22, *p* < 0.01] and impulsive-antisocial [*F*_(2, 690)_ = 384.09, *p* < 0.01] psychopathy dimensions, as well as in psychopathy total score [*F*_(2, 690)_ = 343.71, *p* < 0.01], where heroin users scored the highest, followed by amphetamine users and control participants (*p*s < 0.01). With regards to neurocognitive indices of impulsivity, groups differed in MCQ Overall k index of delay discounting [*F*_(2, 653)_ = 6.66, *p* < 0.01]. Tukey's *post-hoc* comparisons reveal that control participants had lower discounting rates than heroin users. In addition, there were group differences in MCQ Small k index of delay discounting, measuring the temporal discounting rate of small magnitude rewards [*F*_(2, 653)_ = 7.66, *p* < 0.01], where both amphetamine- and heroin users had higher discounting rates than control participants (*p* < 0.05). Please see Tables 1, 2 for participants' characteristics. Table 1 provides descriptive statistics and group differences in demographic and substance use variables. Table 2 provides descriptive statistics and group differences in indices of psychopathy, impulsive choice, and impulsive action.

**Table 1:** Descriptive statistics and group differences in demographic and substance use variables.

	**Controls (1)**	**HDIs (2)**	**ADIs (3)**	***p***	**Contrasts**
*N*	319	183	191	–	–
Age	28.41 (7.64)	31.96 (5.98)	25.61 (5.57)	**0.000**	2 > 1 > 3
Biological sex (N/% male)	169 (53%)	141 (77%)	136 (71.2%)	**0.000**	-
Raven's estimated IQ	109.19 (13.94)	105.20 (12.87)	109.05 (12.68)	**0.003**	1, 3 > 2
Years education	14.51 (2.76)	12.86 (2.55)	13.20 (2.18)	**0.000**	1 > 2, 3
Length of abstinence	–	5.67 (5.57)	2.96 (3.00)	**0.000**	2 > 3
N of symptoms heroin/amphetamine dependence	–	6.20 (0.97)	4.74 (1.76)	**0.000**	2 > 3

*HDIs, heroin dependent individuals; ADIs, amphetamine dependent individuals. Values in bold are significant*.

**Table 2:** Descriptive statistics and group differences in indices of psychopathy, decision-making, and response inhibition.

	**Controls (1)**	**HDIs (2)**	**ADIs (3)**	***p***	**Contrasts**
PCL:SV factor 1	1.52 (1.76)	5.45 (2.75)	3.81 (2.75)	**0.000**	2 > 3 > 1
PCL:SV factor 2	1.81 (2.15)	7.79 (2.74)	6.39 (2.92)	**0.000**	2 > 3 > 1
PCL:SV total score	3.32 (3.46)	13.25 (4.96)	10.20 (4.98)	**0.000**	2 > 3 > 1
IGT net score	4.17 (27.52)	−1.41 (26.09)	0.58 (26.27)	0.069	–
CGT quality of decision-making	0.89 (0.13)	0.86 (0.14)	0.87 (0.14)	0.073	–
CGT risk taking	0.57 (0.15)	0.59 (0.14)	0.59 (0.15)	0.161	–
MCQ overall *k*	−3.66 (1.55)	−3.17 (1.36)	−3.35 (1.46)	**0.001**	2 > 1
MCQ small *k*	−3.18 (1.47)	−2.69 (1.29)	−2.82 (1.38)	**0.001**	2, 3 > 1
BART pumps adjusted average	40.06 (12.99)	39.77 (13.20)	41.05 (14.95)	0.622	–
GNG false alarms	15.15 (9.3.)	17.16 (16.67)	17.35 (9.28)	0.063	–
IMT commission errors	38.17 (14.92)	39.47 (14.41)	39.18 (13.12)	0.568	–
SST 150 ms inhibition	71.68 (21.34)	71.58 (19.94)	71.79 (21.26)	0.995	–

*HDIs, heroin dependent individuals; ADIs, amphetamine dependent individuals. PCL:SV Factor 1, Psychopathy Checklist: Screening Version Factor 1; PCL:SV Factor 2, Psychopathy Checklist: Screening Version Factor 2; PCL:SV Total score, Psychopathy Checklist: Screening Version Total score; MCQ Overall k, MCQ Overall temporal discounting rate; MCQ Small k, MCQ Temporal discounting rate of small magnitude rewards; BART Pumps adjusted average, adjusted average number of pumps on unexploded balloons. Values in bold are significant*.

All main analyses were performed using groups of heroin and amphetamine users, consisting of both mono-dependent, and polysubstance dependent individuals. For detailed participants characteristics across groups of heroin- and amphetamine mono- and polysubstance dependent individuals, please see Supplementary Tables 1, 2.

### Regression Analyses

#### Impulsive Choice

**Figure 1:**
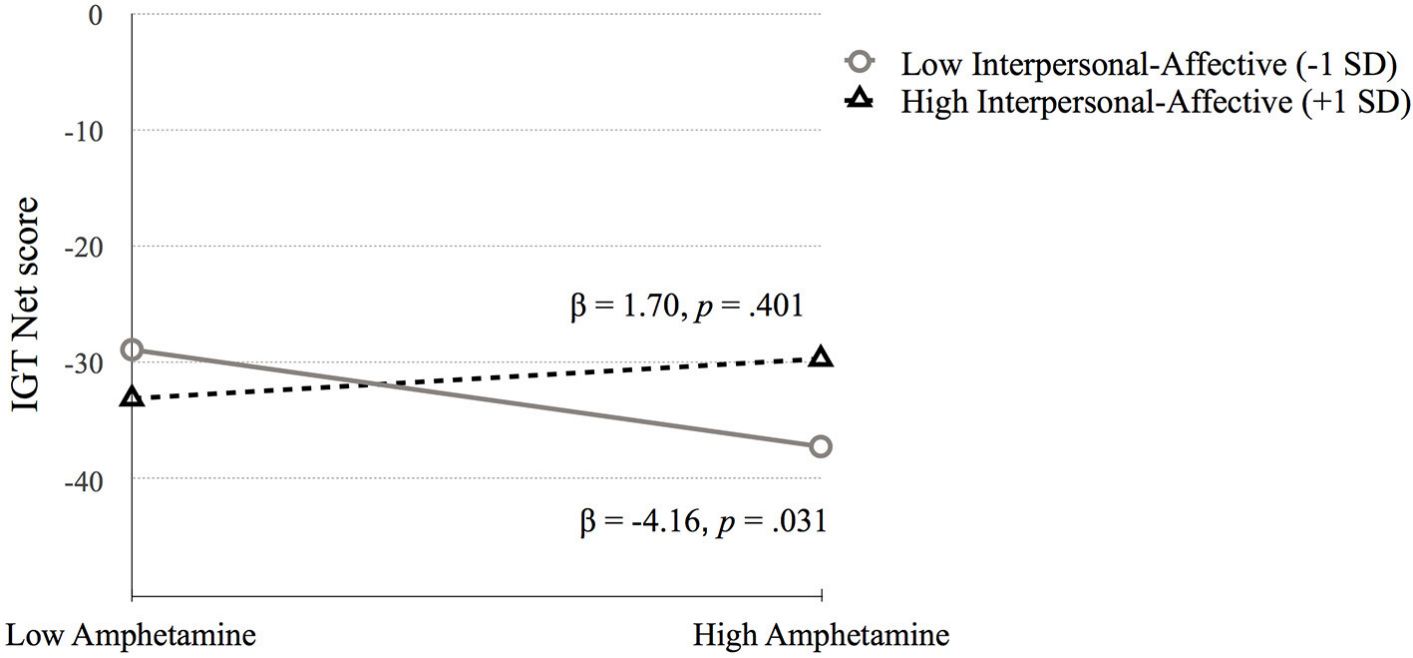
The moderating effect of Interpersonal-affective psychopathy dimension on the association between amphetamine dependence and IGT Net score. Low and high values represent +1.0 and −1.00 *SD* from the mean.

*Iowa Gambling Task* (**IGT Net score**). Step 1 was significant, *F*_(4, 675)_ = 7.25, *p* < 0.001. Higher IGT Net scores were associated with higher IQ (*p* < 0.001) and fewer symptoms of heroin dependence (*p* = 0.042) and amphetamine dependence (*p* = 0.031). Step 2 added the PCL:SV factors to step 1 [*F*_(6, 673)_ = 5.17, *p* < 0.001]. Both heroin (*p* = 0.229) and amphetamine dependence (*p* = 0.221) became nonsignificant, and the psychopathy factors were not significant predictors of IGT Net score. Step 3 added the interaction term between psychopathy and substance dependence, [*F*_(10, 669)_ = 3.61, *p* < 0.001]. The change in *R*^2^ was not significant (*p* = 0.283). The interaction between factor 1 and amphetamine was significant (*p* = 0.044). Probing this interaction using simple slopes analysis revealed that amphetamine dependence symptoms were related to IGT Net score at low levels of Factor 1 (*p* = 0.031) and not at high levels of Factor 1 (*p* = 0.401). Thus, lower Factor 1 scores contribute to the association between amphetamine dependence symptoms and poor performance on IGT Net score, whereas higher Factor 1 scores may serve as a buffer in the association between amphetamine dependence and IGT Net score performance, as indicated by the nonsignificant difference (see Figure 1, Table 3).

**Table 3:** Substance use and psychopathy as predictors of (1) IGT Net score, (2) CGT Quality of decision-making, and (3) CGT Risk taking.

	**IGT net score**				**CGT quality of decision-making**				**CGT risk taking**			
	***B***	***SE B***	**β**	**Δ*R^**2**^***	***B***	***SE B***	**β**	**Δ*R^**2**^***	***B***	***SE B***	**β**	**Δ*R^**2**^***
**Step 1**				0.04^**^				0.03^**^				0.05^**^
Biological sex	−2.13	2.15	−0.04		0.02	0.01	0.08^*^		−0.06	0.01	−0.20^***^	
Raven's estimated IQ	0.34	0.08	0.17^***^		0.00	0.00	0.14^***^		0.00	0.00	−0.02	
Heroin	−2.13	1.05	−0.08^*^		−0.01	0.01	−0.04		0.00	0.01	−0.02	
Amphetamine	−2.21	1.02	−0.08^*^		0.00	0.01	−0.01		0.01	0.01	0.09^*^	
**Step 2**				0.00				0.00				0.01
Biological sex	−1.88	2.32	−0.03		0.02	0.01	0.08		−0.06	0.01	−0.18	
Raven's estimated IQ	0.33	0.08	0.16^***^		0.00	0.00	0.13^**^		0.00	0.00	−0.01	
Heroin	−1.63	1.35	−0.06		0.00	0.01	−0.01		−0.01	0.01	−0.08	
Amphetamine	−1.54	1.26	−0.06		0.00	0.01	0.01		0.01	0.01	0.03	
Factor 1	1.78	1.60	0.07		0.00	0.01	0.01		0.00	0.01	0.02	
Factor 2	−2.41	1.79	−0.09		−0.01	0.01	−0.05		0.02	0.01	0.10	
**Step 3**				0.01				0.00				0.07
Biological sex	−2.20	2.33	−0.04		0.02	0.01	0.07		−0.05	0.01	−0.17	
Raven's estimated IQ	0.34	0.08	0.17		0.00	0.00	0.13^**^		0.00	0.00	−0.01	
Heroin	−2.29	1.55	−0.09		0.00	0.01	0.00		−0.01	0.01	−0.03	
Amphetamine	−1.23	1.34	−0.05		0.00	0.01	0.00		0.00	0.01	0.02	
Factor 1	0.83	1.68	0.03		0.00	0.01	0.01		0.01	0.01	0.06	
Factor 2	−1.65	1.82	−0.06		−0.01	0.01	−0.05		0.01	0.01	0.08	
Heroin X factor 1	0.65	1.51	0.02		0.00	0.01	−0.01		−0.02	0.01	−0.15^*^	
Heroin X factor 2	0.76	1.74	0.03		0.00	0.01	−0.01		0.01	0.01	0.04	
Amphetamine X factor 1	2.93	1.45	0.11^*^		0.00	0.01	0.02		−0.01	0.01	−0.05	
Amphetamine X factor 2	−2.06	1.62	−0.07		0.00	0.01	0.00		0.01	0.01	0.04	

*Biological sex, Male (1), Female (2);*

* *p < 0.05;*

** *p < 0.01;*

*** *p < 0.001*.

**Figure 2:**
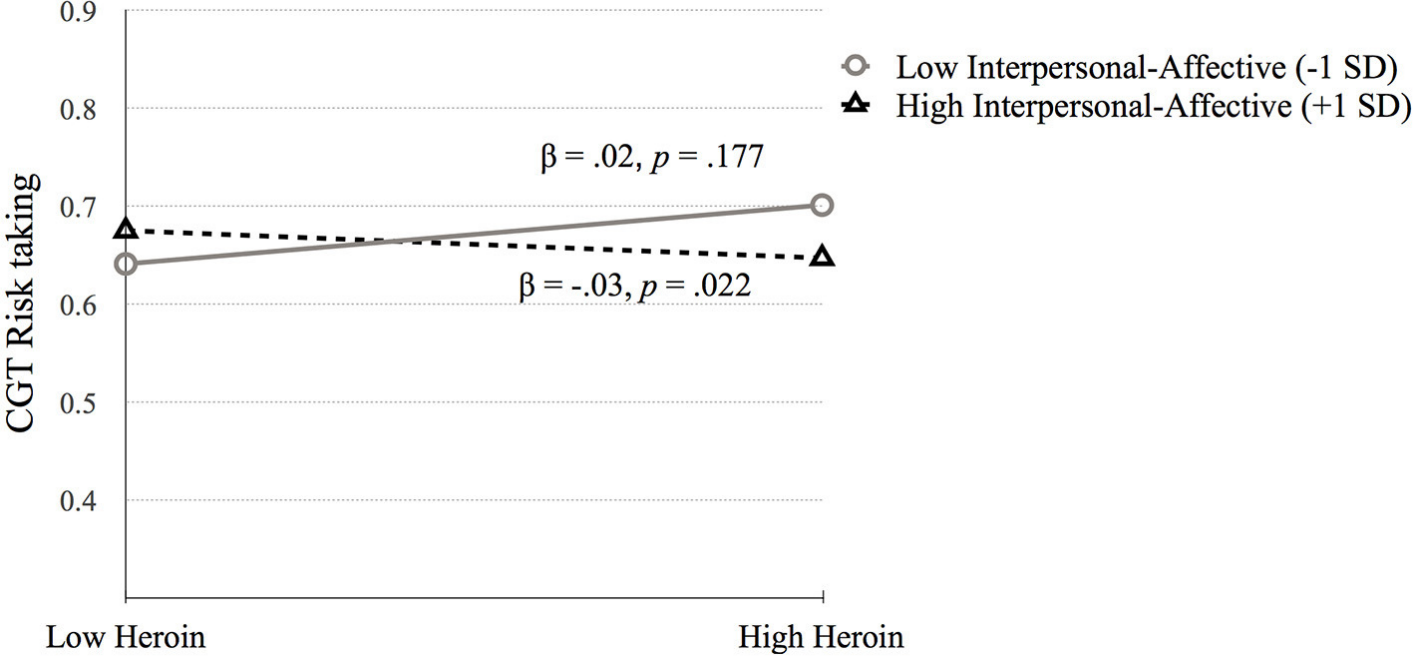
The moderating effect of Interpersonal-affective psychopathy dimension on the association between heroin dependence and CGT Risk taking. Low and high values represent +1.0 and −1.00 *SD* from the mean.

*Cambridge Gambling Task*. **(1) CGT Quality of decision-making**. Step 1 was significant, *F*_(4, 648)_ = 5.03, *p* = 0.001. IQ (*p* < 0.001) and biological sex (*p* = 0.038) were positively related to CGT Quality of decision-making (*p* < 0.001). Step 2 [*F*_(6, 646)_ = 3.46, *p* = 0.002] and step 3 were significant [*F*_(10, 642)_ = 2.13, *p* = 0.020], but no significant variables emerged. Therefore, higher IQ and being female was associated with higher quality of decision-making (see Table 3). **(2) CGT Risk taking**. Step 1 was significant, *F*_(4, 648)_ = 8.53, *p* < 0.001. Being male (*p* < 0.001) and higher amphetamine dependence symptoms (*p* = 0.028) were related to higher CGT Risk taking scores. Step 2 was significant [*F*_(6, 646)_ = 6.30, *p* < 0.001] but no new variables were significant. Step 3 was significant [*F*_(10, 642)_ = 4.67, *p* < 0.001] and *R*^2^ change approached significance (*p* = 0.071). The interaction between heroin dependence and PCL:SV Factor 1 was significant (*p* = 0.009). The simple slopes analysis was significant for high levels of Factor 1 (*p* = 0.022) but not for low levels (*p* = 0.177; See Figure 2). High PCL:SV Factor 1 scores in individuals with more symptoms of heroin dependence was associated with less risky decision-making (see Table 3).

*Monetary Choice Questionnaire*. **(1) MCQ Overall k**. Step 1 was significant, *F*_(4, 660)_ = 5.78, *p* < 0.001. Biological sex (*p* = 0.036) and IQ (*p* = 0.001) were related to MCQ Overall k. Step 2 [*F*_(6, 658)_ = 4.71, *p* = 0.001] and step 3 were significant [*F*_(10, 654)_ = 3.32, *p* < 0.001], but no significant predictors emerged. Therefore, being male and having lower IQ were related to higher delay discounting (see Table 4). **(2) MCQ Small k**. Step 1 was significant, *F*_(4, 660)_ = 5.71, *p* < 0.001. Lower IQ (*p* = 0.004) and higher amphetamine dependence symptoms (*p* = 0.042) were associated with MCQ Small k. Step 2 [*F*_(6, 658)_ = 4.03, *p* = 0.001] and step 3 were significant [*F*_(10, 654)_ = 3.16, *p* = 0.001], but no significant predictors emerged (see Table 4).

**Table 4:** Substance use and psychopathy as predictors of (1) MCQ Overall *k*, (2) MCQ Small *k*, and (3) BART Pumps adjusted average.

	**MCQ overall** ***k***				**MCQ small** ***k***				**BART pumps adjusted average**			
	***B***	***SE B***	**β**	**Δ*R^**2**^***	***B***	***SE B***	**β**	**Δ*R^**2**^***	***B***	***SE B***	**β**	**Δ*R^**2**^***
**Step 1**				0.03^***^				0.03^***^				0.03^**^
Biological sex	−0.25	0.12	−0.08^*^		−0.22	0.12	−0.08^**^		−1.70	1.09	−0.06	
Raven's estimated IQ	−0.01	0.00	−0.13^**^		−0.01	0.00	−0.11		0.15	0.04	0.15^***^	
Heroin	0.09	0.06	0.06		0.10	0.06	0.07		0.11	0.54	0.01	
Amphetamine	0.09	0.06	0.06		0.11	0.06	0.08^*^		0.29	0.52	0.02	
**Step 2**				0.01				0.00				0.00
Biological sex	−0.15	0.13	−0.05		−0.18	0.12	−0.06		−1.65	1.18	−0.06	
Raven's estimated IQ	−0.01	0.00	−0.11^**^		−0.01	0.00	−0.11^**^		0.15	0.04	0.15^***^	
Heroin	−0.02	0.08	−0.01		0.05	0.07	0.03		−0.14	0.69	−0.01	
Amphetamine	0.00	0.07	0.00		0.06	0.07	0.05		0.03	0.64	0.00	
Factor 1	0.08	0.09	0.05		0.01	0.08	0.00		−0.36	0.81	−0.03	
Factor 2	0.13	0.10	0.09		0.09	0.10	0.07		0.76	0.91	0.06	
**Step 3**				0.01				0.01				0.00
Biological sex	−0.15	0.13	−0.05		−0.18	0.12	−0.06		−1.51	1.19	−0.05	
Raven's estimated IQ	−0.01	0.00	−0.11^**^		−0.01	0.00	−0.10^*^		0.15	0.04	0.15^***^	
Heroin	0.03	0.09	0.02		0.07	0.09	0.05		0.05	0.79	0.00	
Amphetamine	0.03	0.08	0.02		0.11	0.07	0.08		−0.01	0.68	0.00	
Factor 1	0.09	0.09	0.06		0.02	0.09	0.02		−0.01	0.85	0.00	
Factor 2	0.11	0.10	0.08		0.07	0.10	0.05		0.48	0.93	0.04	
Heroin X factor 1	−0.02	0.08	−0.02		−0.05	0.08	−0.04		−0.32	0.77	−0.02	
Heroin X factor 2	−0.10	0.10	−0.06		−0.05	0.09	−0.03		−0.18	0.89	−0.01	
Amphetamine X factor 1	0.01	0.08	0.01		−0.01	0.08	0.00		−0.96	0.73	−0.07	
Amphetamine X factor 2	−0.12	0.09	−0.08		−0.15	0.09	−0.10		0.49	0.82	0.03	

*Biological sex = Male (1), Female (2);*

* *p < 0.05;*

** *p < 0.01;*

*** *p < 0.001*.

*Balloon Analog Risk Task* (**BART Pumps adjusted average**). Results of the hierarchical regression analyses with BART Pumps adjusted average are displayed in Table 4. Step 1, which included biological sex, IQ, heroin dependence symptoms, and amphetamine dependence symptoms was significant, *F*_(4, 686)_ = 4.33, *p* = 0.002. IQ was positively related to BART Pumps adjusted average (*p* < 0.001). Step 2 added the psychopathy factors, which was significant, *F*_(6, 684)_ = 29.99, *p* = 0.007. However, no new significant variables emerged. Step 3 added the interaction between the psychopathy factors and substance dependence, which was significant, *F*_(10, 680)_ = 2.02, *p* = 0.029 but no interaction terms were significant. In sum, the only predictor to emerge was IQ, which was positively associated with risk taking (BART Pumps adjusted average).

#### Impulsive Action

**Figure 3:**
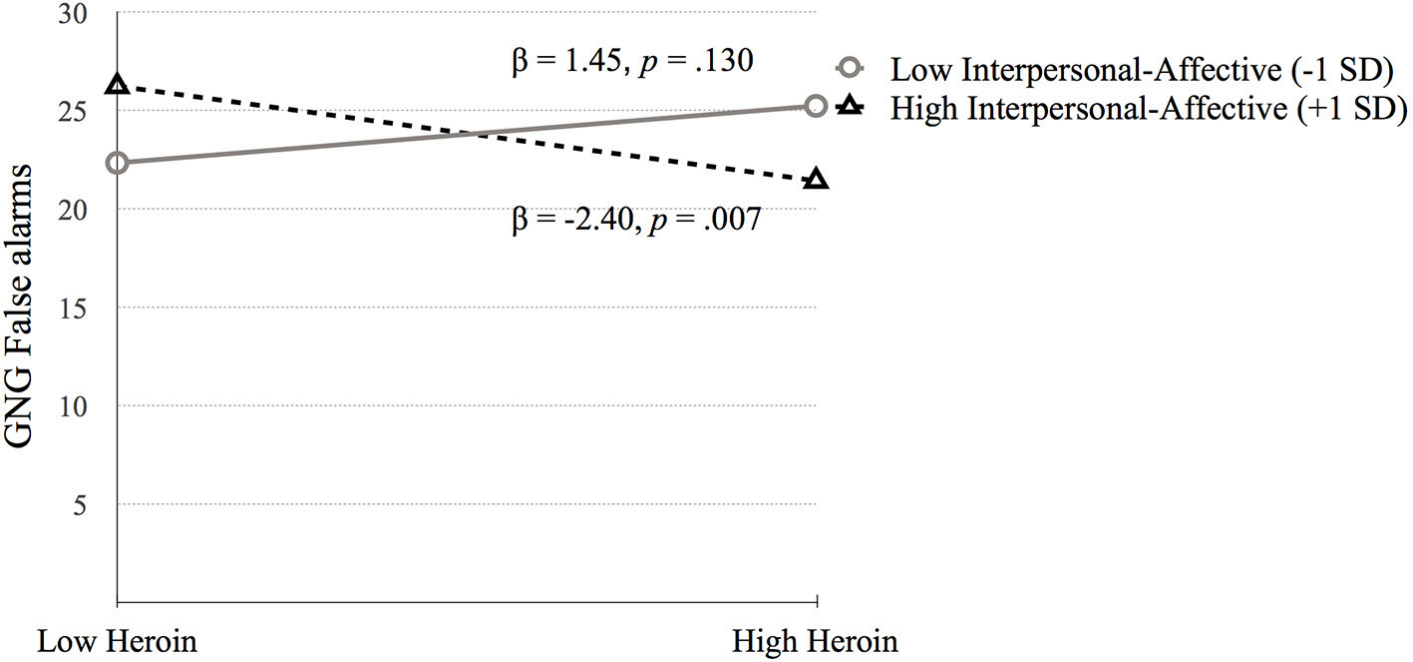
The moderating effect of Interpersonal-affective psychopathy dimension on the association between heroin dependence and GNG False alarms. Low and high values represent +1.0 and −1.00 *SD* from the mean.

*Go/No-Go Task* (**GNG False alarms**). Step 1 was significant, *F*_(4, 683)_ = 6.72, *p* < 0.001. Higher GNG False alarms were associated with lower IQ (*p* = 0.001) and higher amphetamine dependence symptoms (*p* < 0.001). Step 2 added the psychopathy factors to step 1 [*F*_(6, 681)_ = 5.52, *p* < 0.001]. Amphetamine dependence (*p* = 0.221) became non-significant, and Factor 2 was positively associated with GNG False alarms (*p* = 0.021). Step 3 added the interaction terms between psychopathy and substance dependence [*F*_(10, 677)_ = 5.04, *p* < 0.001] but the change in *R*^2^ was not significant (*p* = 0.573). The interactions between heroin dependence and Factor 1 (*p* = 0.003) and Factor 2 (*p* < 0.001) were significant. In addition, the interaction between amphetamine dependence and Factor 2 was significant (*p* = 0.041). Each of these interactions were probed using simple slopes analysis, which revealed that GNG False alarms performance was related to high heroin dependence symptoms for those with high Factor 1 scores (*p* = 0.031; See Figure 3; Table 5).

**Table 5:** Substance use and psychopathy as predictors of (1) GNG False alarms, (2) IMT Commission errors, and (3) SST 150 ms inhibition.

	**GNG false alarms**				**IMT commission errors**				**SST 150 ms inhibition**			
	***B***	***SE B***	**β**	**Δ*R^**2**^***	***B***	***SE B***	**β**	**Δ*R^**2**^***	***B***	***SE B***	**β**	**Δ*R^**2**^***
**Step 1**				0.04^***^				0.03^***^				0.04^**^
Biological sex	1.30	0.93	0.05		−0.86	1.14	−0.03		−4.45	1.69	−0.10	
Raven's estimated IQ	−0.12	0.03	−0.13^**^		−0.19	0.04	−0.18^***^		−0.04	0.06	−0.03^**^	
Heroin	0.65	0.46	0.06		−0.19	0.56	−0.01		0.17	0.83	0.01	
Amphetamine	1.60	0.44	0.14^***^		0.82	0.54	0.06		0.33	0.80	0.02	
**Step 2**				0.01^*^				0.00				0.00
Biological sex	1.77	1.01	0.07		−0.14	1.23	−0.01		−5.17	1.83	−0.12	
Raven's estimated IQ	−0.10	0.03	−0.12^**^		−0.18	0.04	−0.17^***^		−0.05	0.06	−0.03^**^	
Heroin	−0.16	0.58	−0.01		−0.93	0.71	−0.06		0.44	1.06	0.02	
Amphetamine	0.83	0.54	0.07		0.24	0.66	0.02		0.36	0.99	0.02	
Factor 1	−0.33	0.68	−0.03		0.61	0.83	0.04		−1.59	1.23	−0.08	
Factor 2	1.79	0.77	0.15^*^		0.88	0.95	0.06		0.85	1.41	0.04	
**Step 3**				0.02^**^				0.01				0.01
Biological sex	1.83	1.00	0.08		−0.27	1.23	−0.01		−4.84	1.82	−0.11	
Raven's estimated IQ	−0.10	0.03	−0.12^**^		−0.17	0.04	−0.16^***^		−0.05	0.06	−0.03^**^	
Heroin	−0.48	0.66	−0.04		−1.08	0.82	−0.08		−0.35	1.21	−0.02	
Amphetamine	0.66	0.57	0.06		0.44	0.71	0.03		0.30	1.05	0.01	
Factor 1	0.02	0.71	0.00		0.34	0.87	0.02		−0.82	1.29	−0.04	
Factor 2	1.76	0.78	0.15^*^		1.15	0.97	0.08		0.40	1.43	0.02	
Heroin X factor 1	−1.93	0.64	−0.17^**^		−0.64	0.80	−0.04		−0.03	1.18	0.00	
Heroin X factor 2	2.61	0.74	0.21^***^		0.72	0.92	0.05		1.69	1.37	0.07	
Amphetamine X factor 1	−0.60	0.61	−0.05		1.20	0.75	0.08		−3.05	1.11	−0.15^**^	
Amphetamine X factor 2	1.40	0.69	0.11^*^		−1.18	0.85	−0.08		2.73	1.26	0.12^*^	

*Biological sex = Male (1), Female (2);*

* *p < 0.05;*

** *p < 0.01;*

*** *p < 0.001*.

**Figure 4:**
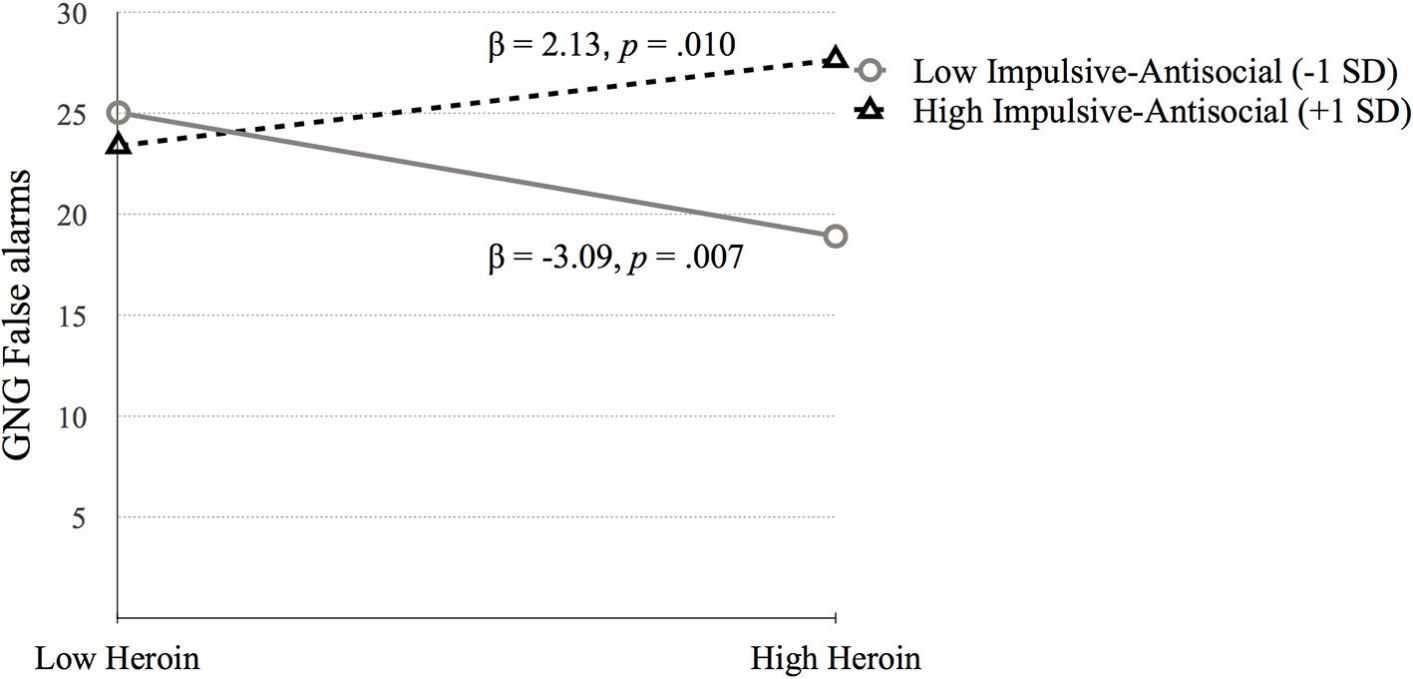
The moderating effect of Impulsive-antisocial psychopathy dimension on the association between heroin dependence and GNG False alarms. Low and high values represent +1.0 and −1.00 *SD* from the mean.

Simple slopes analysis testing the interaction between heroin dependence and Factor 2 indicated that higher GNG False alarms scores were related to high heroin dependence symptoms at high Factor 2 scores (*p* = 0.010), while lower GNG False alarms scores were related to high heroin at low Factor 2 scores (*p* = 0.007; see Figure 4).

**Figure 5:**
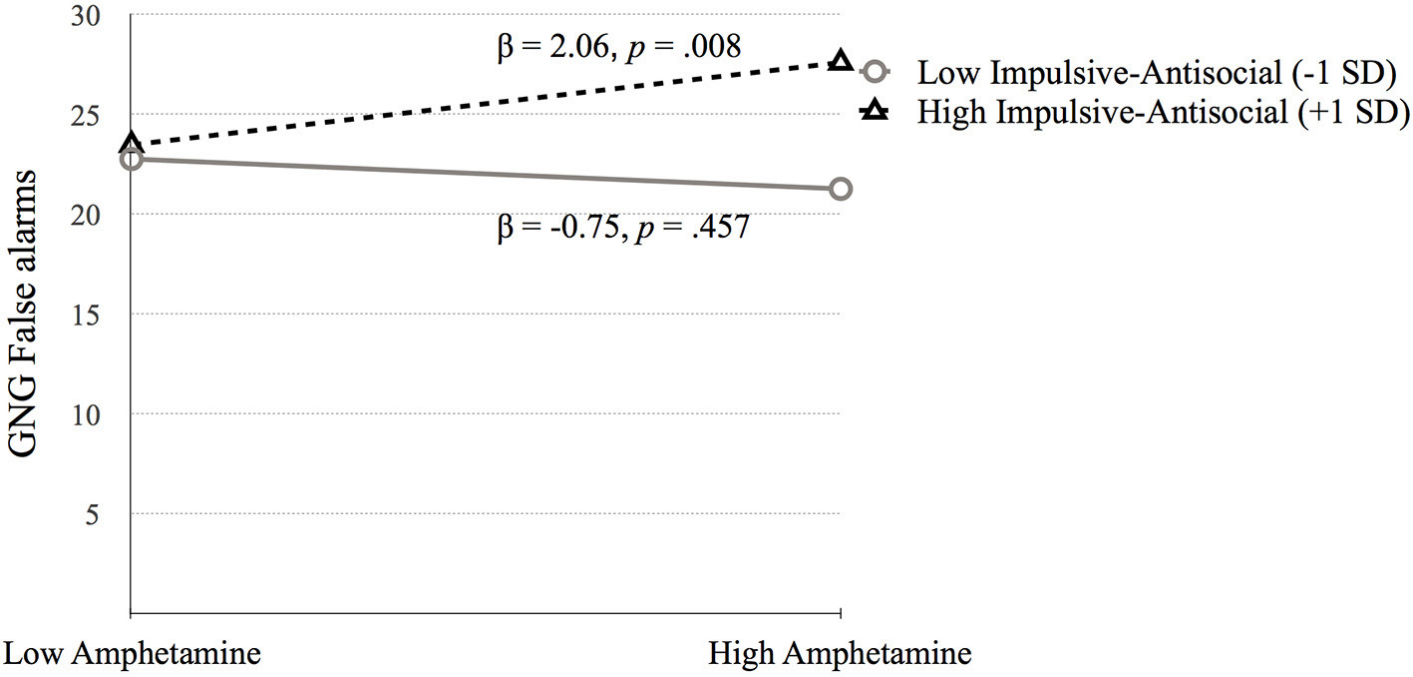
The moderating effect of Impulsive-antisocial psychopathy dimension on the association between amphetamine dependence and GNG False alarms. Low and high values represent +1.0 and 1.00 *SD* from the mean.

The simple slopes model for the interaction term between amphetamine and Factor 2 suggests that higher scores of GNG False alarms are related to high amphetamine dependence symptoms at high factor 2 scores (see Figure 5).

*Immediate Memory Task* (**IMT Commission errors**). Step 1 was significant, *F*_(4, 693)_ = 6.16, *p* < 0.001, which showed that higher IMT Commission errors were associated with lower IQ (*p* = 0.001). Step 2 [*F*_(6, 691)_ = 4.60, *p* < 0.001] and step 3 were significant [*F*_(10, 687)_ = 3.21, *p* < 0.001], but no significant variables emerged. Thus, lower IQ was related to higher errors of commission (see Table 5).

**Figure 6:**
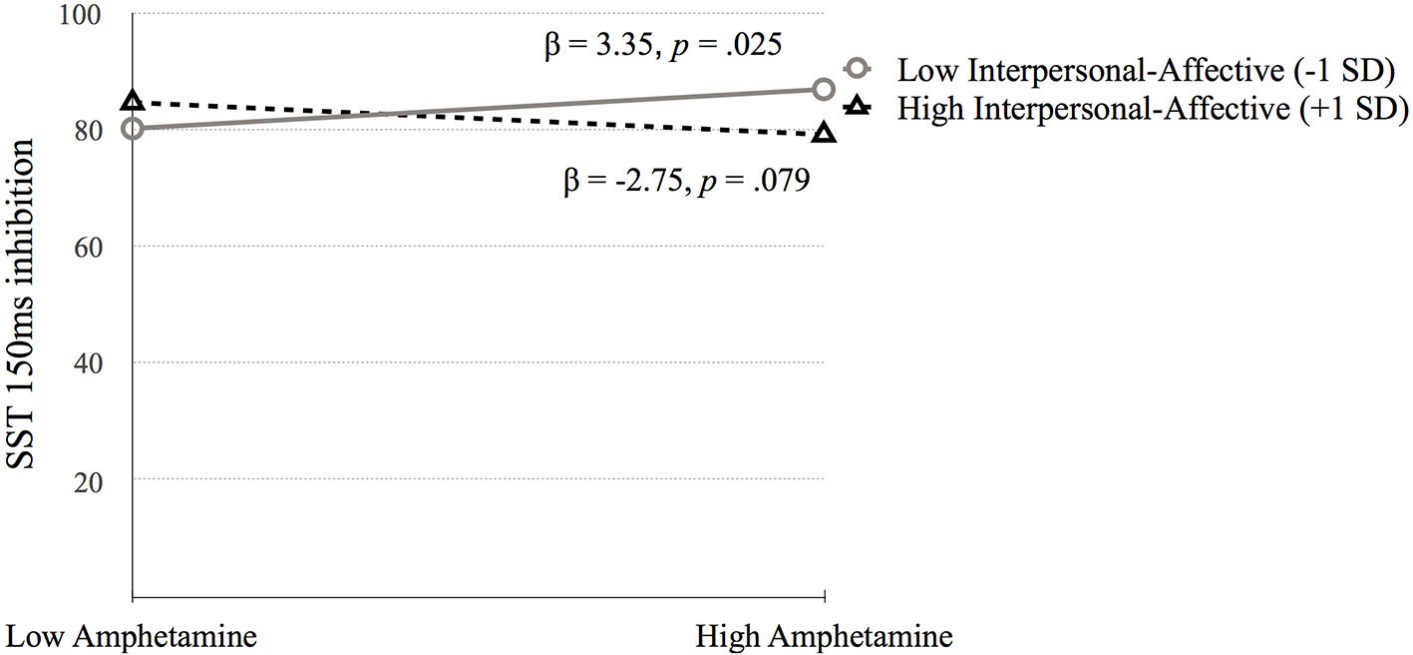
The moderating effect of Interpersonal-affective psychopathy dimension on the association between amphetamine dependence and SST 150 ms inhibition. Low and high values represent +1.0 and −1.00 *SD* from the mean.

**Figure 7:**
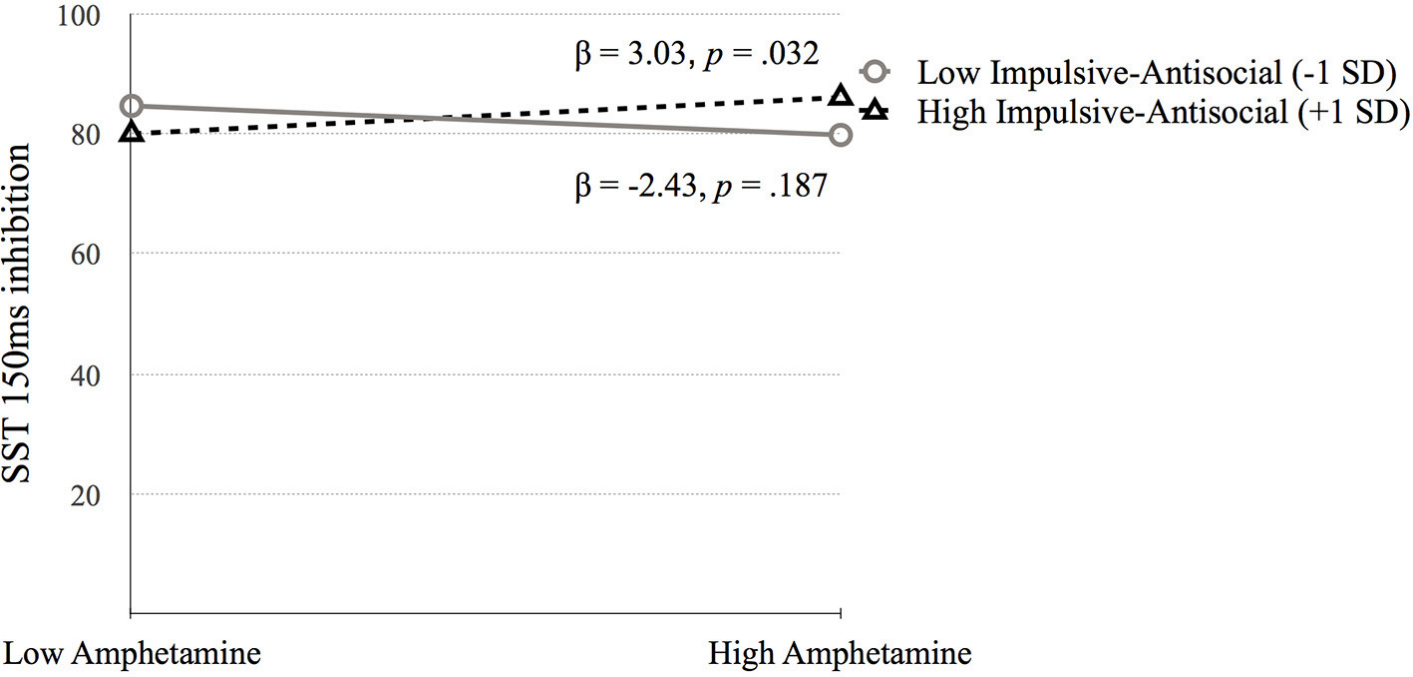
The moderating effect of Impulsive-antisocial psychopathy dimension on the association between amphetamine dependence and SST 150 ms inhibition. Low and high values represent +1.0 and −1.00 *SD* from the mean.

*Go Stop Task* (**SST 150ms inhibition**). Table 5 presents the results of the hierarchical regression. Neither step 1 [*F*_(4, 688)_ = 2.11, *p* = 0.078) nor step 2 [*F*_(6, 686)_ = 1.68, *p* = 0.122] were significant. Step 3, which included the interaction terms between psychopathy and SUD was significant, *F*_(10, 682)_ = 2.05, *p* = 0.027. SST 150 ms inhibition was associated with amphetamine dependence when moderated by Factor 1 (*p* = 0.006) and Factor 2 (*p* = 0.031). Factor 1 of psychopathy moderated the association between amphetamine dependence and SST 150 ms inhibition at low levels of Factor 1 (*p* = 0.025) but not at high levels of Factor 1 (*p* = 0.079; Figure 6). In contrast, Factor 2 moderated the relation between amphetamine dependence and SST 150 ms inhibition at high levels of Factor 2 (*p* = 0.032) but not at low levels of Factor 2 (*p* = 0.187; Figure 7). Thus, amphetamine dependence was related to higher SST 150 ms inhibition scores (i.e., lower impulsivity) when individuals had either low Factor 1 psychopathy scores or high Factor 2 psychopathy scores. This result highlights that psychopathy factors can differentially serve as both risk and protective factors for neurocognitive function in people with amphetamine dependence.

## Discussion

The aims of the present study were to examine the effects of psychopathy and its two dimensions (interpersonal-affective and impulsive-antisocial) on the relationships between dependence on different classes of drugs (stimulants and opioids) and distinct neurocognitive domains of impulsivity (impulsive choice/decision-making and impulsive action/response inhibition). Our findings suggest that the two dimensions of psychopathy had both common and unique moderating effects on decision-making and response inhibition in individuals dependent on stimulants or opiates.

Within the domain of impulsive choice, our results demonstrate that the interpersonal-affective dimension of psychopathy (Factor 1) moderates the associations between quality of decision-making, risk-taking, and dependence in a similar manner for opiates and stimulants. Specifically, lower risk taking on the CGT was predicted by the combination of more symptoms of heroin dependence and high scores on the interpersonal-affective dimension of psychopathy (PCL:SV Factor 1). With few exceptions (78, 79), previous studies conducted separately with psychopathic individuals (80–84, 95) and with opioid dependent individuals (47–49, 56, 61, 109) report that both groups are characterized by riskier and less advantageous decision-making. To our knowledge, only one study to date has examined the effects of co-occurring psychopathy and opioid dependence on decision-making. Vassileva et al. (86) reported that psychopathic heroin users were characterized by more impaired decision-making than non-psychopathic heroin users, suggesting that psychopathy may exacerbate decision-making deficits in opiate dependent individuals. However, Vassileva et al. (86) considered psychopathy as a unitary categorical construct rather than examining its underlying dimensions, therefore it remained unclear which features of psychopathy were associated with more impaired decision-making in heroin users and whether some psychopathic traits may act as a buffer against disadvantageous and risky decision-making within the context of opioid addiction. The current study builds upon previous findings and indicates that the interpersonal-affective features of psychopathy (Factor 1) may paradoxically play a protective role and reduce the predisposition toward risky decision-making in heroin users.

Findings related to the utility of the two psychopathy dimensions for predicting decision-making in individuals with amphetamine dependence were somewhat consistent with those observed among heroin users. Specifically, results revealed that poor performance on the IGT was predicted by the combination of more symptoms of amphetamine dependence and lower scores on the PCL:SV Factor 1, suggesting that the interpersonal-affective dimension of psychopathy may have similarly protective effect on decision-making in amphetamine dependent individuals as it does in heroin dependent individuals. Interestingly, these results reveal that although the PCL:SV Factor 1 might have common protective effect on reward-based decision-making in both opiate and stimulant dependent individuals, it affects different types of decision-making in heroin and amphetamine users. Specifically, it was related to decision-making under ambiguity in amphetamine users, whereas it was associated with decision-making under risk in heroin users (58, 110). Therefore, our data suggest that the interpersonal-affective dimension of psychopathy may be a key factor that may account for the differential neurocognitive impulsivity profiles observed in individuals dependent on opiates vs. stimulants. Our findings are also consistent with previous studies that have found that the interpersonal-affective traits of psychopathy were either unrelated or negatively related to overall decision-making deficits (80, 95, 96). Unlike previous studies, which fail to address the unique effects of different dimensions of psychopathy on decision-making in substance users, our study was focused on the predictive utility of the two psychopathy dimensions on the quality of decision-making in different types of SUDs and on different reward-based decision-making tasks. Our findings reveal that the interpersonal-affective rather than the impulsive-antisocial dimension of psychopathy contributes significantly to intact decision-making in the context of both opioid and stimulant addictions, and appears to be the key factor of psychopathy that moderates reward-based decision-making in individuals with SUDs, regardless of specific drug class.

Within the domain of impulsive action, both the interpersonal-affective and the impulsive-antisocial dimensions of psychopathy predicted varying levels of response disinhibition among individuals dependent on opioids or stimulants. High scores on the impulsive-antisocial Factor 2 of psychopathy exacerbated response inhibition deficits on the Go/No-Go task in both amphetamine- and heroin users. These results are in line with previous findings from studies conducted separately with psychopathic individuals (87, 88, 97) and individuals dependent on stimulants (28, 31, 44–46) and/or opioids (31, 47–49), suggesting that psychopathy and dependence on both classes of drugs are related to poor response inhibition. Some studies on psychopathy have also implicated specifically the impulsive-antisocial dimension of psychopathy as the key factor underlying the response inhibition deficits observed in psychopathic individuals (97, 98, 111). Our findings suggest that increased levels of impulsive-antisocial psychopathic traits in the context of addiction may exert additive effects on the already compromised response inhibition performance in substance users.

In contrast, the interpersonal-affective (Factor 1) dimension of psychopathy had differential effects on response inhibition in individuals dependent on opiates vs. stimulants, such that it exacerbated the response inhibition deficits in amphetamine dependent individuals, whereas it was related to better response inhibition in heroin dependent individuals. These results are in line with studies reporting opposite relationships between trait impulsivity and neurocognitive impulsivity in heroin and amphetamine users, where increased trait impulsivity was associated with worse response inhibition in amphetamine dependent individuals, but with better response inhibition in heroin dependent individuals (7). There are reports that the interpersonal-affective dimension of psychopathy is related to superior response inhibition among psychopathic individuals (92, 97, 98). However, research findings to date are equivocal, with some studies finding positive associations between interpersonal-affective psychopathic traits and response inhibition (97, 98), while others have failed to find any relationships or have reported negative relationships (112, 113). These conflicting findings may be explained at least partially by the highly heterogenous samples across studies, e.g., criminal offenders (97, 98) vs. students (112, 113). In addition, inconsistencies between studies could be due to differences in the assessment of psychopathy [interview-based measures such as the PCL (97, 98) vs. self-report measures (112, 113)], differences in the paradigms used to assess response inhibition which may lead to task-specific effects, and the lack of control for concurrent SUDs. Our results are limited to opiate and stimulant use disorders and are focused on the effects of specific combinations between dependence on different classes of drugs (stimulants and opioids) and psychopathy dimensions as predictors of response inhibition. Our findings suggest that drug of choice may interact uniquely with the interpersonal-affective traits of psychopathy and result either in better response inhibition in heroin dependent individuals, or poor response inhibition in amphetamine dependent individuals. It is important to note that in the current sample the levels of the interpersonal-affective dimension of psychopathy were significantly higher among heroin users than in amphetamine users. Therefore, it is possible that more pronounced interpersonal-affective traits can contribute to intact response inhibition, irrespective of the unique effects of the drug of choice. In addition, our results suggest that the effects of the PCL:SV Factor 1 on response inhibition might be task dependent in heroin and amphetamine users. That is, in amphetamine dependent individuals the interpersonal-affective psychopathy dimension predicted diminished ability to cancel an already initiated response as measured by the Go Stop task, whereas in heroin dependent individuals it was associated with the ability to inhibit a prepotent motor response that has not been triggered yet as measured by the Go/No-Go task.

One surprising finding was that the combination of more symptoms of amphetamine dependence and higher impulsive-antisocial features of psychopathy predicted increased inhibitory control on the Stop Signal Task. This indicates that the impulsive-antisocial dimension of psychopathy had differential effects on different tasks of impulsive action in amphetamine users, facilitating the cancellation of an already triggered prepotent motor reaction, while exacerbating the difficulties in the ability to inhibit a dominant response that has not been triggered yet. These findings are in line with previous studies, which have suggested that distinct impulsive action tasks (e.g., Go/No-Go, Stop Signal Tasks) reflect independent cognitive processes, such as “controlled top-down inhibition” in Stop Signal Tasks vs. “automatic bottom-up inhibition” in Go/No-Go Tasks (114) that are mediated by different neural circuits (115–117). Therefore, our results provide further evidence for the distinction between different types of neurocognitive impulsivity and the need to evaluate them separately when examining the specific profiles of neurocognitive impairments in individuals with different types of psychopathology.

In summary, our findings suggest that psychopathy dimensions could play an important role in explaining the decision-making and response inhibition deficits commonly observed in substance users, which may have important clinical implications. First, our results suggest that although screening for psychopathy is rarely conducted in SUDs treatment programs, it would provide valuable information, which could facilitate the development of more personalized interventions aimed at decreasing the negative treatment outcomes related to specific personality and neurocognitive risk factors. For example, the development and implementation of treatment interventions targeting the impulsive-antisocial aspects of psychopathy could be of particular importance when working with substance users with impaired response inhibition and higher scores on PCL:SV Factor 2. On the other hand, detecting higher interpersonal-affective psychopathic traits could be a resource for improving the quality of decision-making among substance users. Such interventions could potentially help reduce relapse rates in substance users, which are commonly predicted by higher response disinhibition and impaired decision-making (32–37) and may be significantly influenced by certain personality characteristics. Nevertheless, our findings require further investigation and replication in samples with other types of SUDs (e.g., alcohol-, cannabis use disorders) and at different stages of the addiction cycle. In addition, other personality profiles could be tested as predictors of neurocognitive impairments among substance users, which could lead to the development of enriched variety of interventions and therapeutic techniques that are not uniformly applied among substance users, but are rather tailored to the individual characteristics of the highly heterogeneous group of substance users.

## Limitations and Future Directions

A few important limitations need to be considered. First, our findings are specific to the protracted abstinence stage of opiate and stimulant addiction and should not be generalized to other stages of the addiction cycle or to other types of SUDs. Future studies should examine whether psychopathy dimensions have similar moderating effects on decision-making and response inhibition in individuals dependent on other classes of drugs. Second, our findings were based on the traditional two-factor model of psychopathy and should be examined with other models, such as the 4-facet model, which includes interpersonal, affective, lifestyle and antisocial dimensions (118) and has been proposed to provide a more sensitive approach in studying the associations between psychopathy and other variables (119). Future studies should also examine whether psychopathy dimensions predict neurocognitive impairments differently in mono- vs. polysubstance-dependent individuals. Third, we used a community sample of Bulgarian substance users. Therefore, caution is warranted in generalizing the conclusions of our findings before they are replicated cross-culturally. Another limitation of the current study is that there was no comprehensive evaluation of co-occurring psychiatric disorders, that are commonly comorbid with SUDs, such as affective, neurodevelopmental and personality disorders. Future studies could examine more thoroughly the possible effects of comorbid psychopathology on the relationships between psychopathy dimensions and neurocognitive impulsivity among substance users. Finally, statistical tests were uncorrected for multiple comparisons and conducted using an alpha level of 0.05. An alternative would be to apply the Bonferroni correction, which may change the interpretation of some results. However, this method could be overly conservative when conducting multiple regressions, resulting in a type I error rate much smaller than the desired alpha, therefore all tests were conducted using an unadjusted alpha (120).

## Conclusion

In summary, our results reveal that distinct dimensions of psychopathy have both common and unique moderating effects on neurocognitive impulsivity in individuals in protracted abstinence who are dependent on different classes of drugs (stimulants vs. opiates). In heroin dependent individuals the interpersonal-affective features of psychopathy may play a protective role on both response inhibition and decision-making, whereas in amphetamine dependent individuals lower scores on this dimension of psychopathy were associated with poor decision-making and superior response inhibition. These findings suggest that the interpersonal-affective features of psychopathy have similar effects on decision-making and opposite effects on response inhibition in heroin- and amphetamine dependent individuals. In contrast, higher scores on the impulsive-antisocial dimension of psychopathy predicted response disinhibition in both heroin- and amphetamine dependent individuals, suggesting that the PCL:SV Factor 2 had common deleterious effects on the ability to inhibit prepotent motor responses in people with SUDs, regardless of drug of choice. In addition, impulsive-antisocial psychopathic traits were uniquely related to increased ability to cancel an already initiated response in amphetamine dependent individuals. Overall, our results suggest that not psychopathy *per se*, but rather the interaction between its two dimensions and dependence on specific classes of drugs may lead to either deficient or superior response inhibition and decision-making performance in individuals with SUDs in protracted abstinence.

## Data Availability Statement

The raw data supporting the conclusions of this article will be made available by the authors, without undue reservation.

## Ethics Statement

All subjects gave written informed consent in accordance with the Declaration of Helsinki. The study protocol was approved by the Institutional Review Boards of Virginia Commonwealth University and the Medical University in Sofia on behalf of the Bulgarian Addictions Institute.

## Author Contributions

EP, NT, and JV conceived the study. NT performed the statistical analyses and drafted the analysis and results sections. EP drafted the Introduction, Methods, and Discussion sections. EP, KB, DN, and GV collected and managed the data. JV supervised the data collection and analyses and drafted portions of the manuscript. All authors discussed the results and contributed to the final manuscript.

## Conflict of Interest

GV has ownership interests in the Bulgarian Addictions Institute, where data collection took place. The remaining authors declare that the research was conducted in the absence of any commercial or financial relationships that could be construed as a potential conflict of interest.
