# Supplementary material for: Effects of Psychopathy on Neurocognitive Domains of Impulsivity in Abstinent Opiate and Stimulant Users
Source: Front Psychiatry. 2021 Jun 9;12:660810. doi: 10.3389/fpsyt.2021.660810 (PMC8219927; doi:10.3389/fpsyt.2021.660810)
Supplement: Supplementary file 1 [file Table_1.docx]

Supplementary Material

Supplementary Table 1. Descriptive statistics and group differences in demographic and substance use variables in heroin- and amphetamine mono- and polysubstance dependent individuals.

|  | Controls  (1) | mHDIs  (2) | PHDIs  (3) | mADIs  (4) | pADIs  (5) | p | Contrasts |
| --- | --- | --- | --- | --- | --- | --- | --- |
| N | 319 | 112 | 71 | 115 | 76 | - | - |
| Age | 28.41 (7.64) | 32.38 (6.09) | 31.29 (5.79) | 25.65 (5.64) | 25.55 (5.50) | **.000** | 2, 3 > 1, 4, 5 |
| Biological sex (N/% male) | 169 (53%) | 82 (73.2%) | 59 (83.1%) | 78 (67.8%) | 58 (76.3%) | **.000** | - |
| Raven’s estimated IQ | 109.19 (13.94) | 105.06 (12.54) | 105.41 (13.46) | 108.46 (12.02) | 109.95 (13.66) | **.015** | 1 > 2 |
| Years education | 14.51 (2.76) | 13.07 (2.56) | 12.51 (2.50) | 13.11 (2.22) | 13.34 (2.13) | **.000** | 1 > 2, 3, 4, 5 |
| Length of abstinence | - | 6.74 (5.69) | 3.91 (4.86) | 2.99 (2.86) | 2.91 (3.22) | .524 | - |
| N of symptoms heroin/amphetamine dependence | - | 6.04 (1.03) | 6.20 (1.39) | 4.37 (1.85) | 5.14 (1.66) | **.000** | 2, 3 > 4, 5  5 > 4 |

*Note*. mHDIs = heroin mono-dependent individuals; pHDIs = heroin polysubstance-dependent individuals; mADIs = amphetamine mono-dependent individuals; pADIs = amphetamine polysubstance-dependent individuals.
