# Supplementary material for: Effects of Psychopathy on Neurocognitive Domains of Impulsivity in Abstinent Opiate and Stimulant Users
Source: Front Psychiatry. 2021 Jun 9;12:660810. doi: 10.3389/fpsyt.2021.660810 (PMC8219927; doi:10.3389/fpsyt.2021.660810)
Supplement: Supplementary file 2 [file Table_2.docx]

Supplementary Material

Supplementary Table 2. Descriptive statistics and group differences in indices of psychopathy, decision-making and response inhibition in heroin- and amphetamine mono- and polysubstance dependent individuals.

|  | Controls  (1) | mHDIs  (2) | pHDIs  (3) | mADIs  (4) | pADIs  (5) | p | Contrasts |
| --- | --- | --- | --- | --- | --- | --- | --- |
| PCL:SV Factor 1 | 1.52 (1.76) | 4.98 (2.69) | 6.20 (2.68) | 3.63 (2.83) | 4.08 (2.62) | **.000** | 1 < 2, 3, 4, 5  4 < 2, 3  2 < 3 |
| PCL:SV Factor 2 | 1.81 (2.15) | 7.28 (2.77) | 8.59 (2.52) | 5.92 (2.95) | 7.11 (2.73) | **.000** | 1 < 2, 3, 4, 5  4 < 1, 2, 3  3 > 1, 2, 4, 5 |
| PCL:SV Total score | 3.32 (3.46) | 12.27 (4.92) | 14.79 (4.64) | 9.56 (5.16) | 11.17 (4.54) | **.000** | 1 < 2, 3, 4, 5  2 > 4  3 > 1, 2, 3, 4 |
| IGT Net score | 4.17 (27.52) | 0.92 (27.89) | -5.22 (22.55) | 2.29 (25.08) | -2.03 (27.97) | .069 | - |
| CGT Quality of decision-making | 0.89 (0.13) | 0.86 (0.15) | 0.86 (0.13) | 0.87 (0.14) | 0.87 (0.13) | .259 | - |
| CGT Risk Taking | 0.57 (0.15) | 0.57 (0.13) | 0.61 (0.15) | 0.58 (0.15) | 0.60 (0.14) | .769 | - |
| MCQ Overall *k* | -3.66  (1.55) | -3.23 (1.36) | -3.07 (1.36) | -3.26 (1.48) | -3.48 (1.42) | **.006** | 3 > 1 |
| MCQ Small *k* | -3.18  (1.47) | -2.71 (1.27) | -2.65 (1.34) | -2.76 (1.38) | -2.92 (1.39) | **.003** | 2, 3 > 1 |
| BART Pumps adjusted average | 40.06 (12.99) | 40.21 (13.46) | 39.07 (12.85) | 40.45 (15.17) | 41.96 (14.66) | .769 | - |
| GNG False alarms | 15.15  (9.3) | 16.05 (9.94) | 18.80 (23.31) | 17.32 (8.32) | 17.40 (10.65) | .095 | - |
| IMT Commission errors | 38.17 (14.92) | 37.80 (14.16) | 42.03 (14.51) | 40.79 (13.03) | 36.75 (12.97) | .073 | - |
| SST 150ms inhibition | 71.68 (21.34) | 72.71 (19.06) | 69.78 (21.29) | 69.47 (22.57) | 75.26 (18.74) | .366 | - |

*Note*. mHDIs = heroin mono-dependent individuals; pHDIs = heroin polysubstance-dependent individuals; mADIs = amphetamine mono-dependent individuals; pADIs = amphetamine polysubstance-dependent individuals. PCL:SV Factor 1 = Psychopathy Checklist: Screening Version Factor 1; PCL:SV Factor 2 = Psychopathy Checklist: Screening Version Factor 2; PCL:SV Total score = Psychopathy Checklist: Screening Version Total Score; MCQ Overall k = MCQ Overall Temporal Discounting Rate; MCQ Small k = MCQ temporal discounting rate of small magnitude rewards; BART Pumps adjusted average = adjusted average number of pumps on unexploded balloons;
